# Autophagy Mediates Leptin-Induced Migration and ERK Activation in Breast Cancer Cells

**DOI:** 10.3389/fcell.2021.644851

**Published:** 2021-03-08

**Authors:** Alin García-Miranda, Karen Aylín Solano-Alcalá, José Benito Montes-Alvarado, Arely Rosas-Cruz, Julio Reyes-Leyva, Napoleón Navarro-Tito, Paola Maycotte, Eduardo Castañeda-Saucedo

**Affiliations:** ^1^Laboratorio de Biología Celular del Cáncer, Facultad de Ciencias Químico Biológicas, Universidad Autónoma de Guerrero, Chilpancingo, Mexico; ^2^Centro de Investigación Biomédica de Oriente, Instituto Mexicano del Seguro Social, Puebla, Mexico; ^3^Instituto Tecnológico Superior de Coatzacoalcos, Coatzacoalcos, Mexico; ^4^CONACYT-Centro de Investigación Biomédica de Oriente, Instituto Mexicano del Seguro Social, Puebla, Mexico

**Keywords:** leptin, autophagy, breast cancer, proliferation, migration, ERK

## Abstract

Autophagy is an intracellular recycling process active in eukaryotic cells that involves the formation of an autophagosome which delivers cytoplasmic components to the lysosome for degradation. This process occurs at low rates under basal conditions, but it can be induced by diverse types of stress such as starvation, hypoxia, metabolic disorders or in response to hormones, including leptin. Leptin is considered a pro-tumorigenic protein whose circulating levels have been related to bad prognosis in obese breast cancer patients. It has been recently demonstrated that leptin can induce autophagy in cancer cell lines from different tissues, suggesting that autophagy could modulate the pro-tumorigenic effects associated with leptin. In this study, the role of autophagy in leptin-induced proliferation, migration, apoptosis and ERK phosphorylation in breast cancer cell lines was evaluated. Although leptin differentially induced autophagy in the breast cancer cell lines tested, autophagy inhibition reduced leptin-induced cell proliferation in MCF7 cells and decreased cell migration, ERK activation, and impaired morphological changes in both cell lines. Our data demonstrates an important role for basal autophagy or leptin-induced autophagy in leptin-induced migration and ERK phosphorylation in breast cancer cell lines, suggesting a potential use for the inhibition of autophagy in breast cancer associated with obesity.

## Introduction

Macroautophagy, hereafter referred to as autophagy, is an intracellular recycling process that occurs constitutively in all eukaryotic cells. This process is mainly characterized by the formation of double membrane structures known as autophagosomes, which engulf cytoplasmic components, protein aggregates, or damaged organelles for their subsequent degradation after fusion with the lysosome (Wen and Klionsky, 2020). Autophagy is active at basal levels, but it can be induced upon exposure to stress conditions such as starvation, intracellular damage, metabolic stress, or hypoxia to assist in the maintenance of cellular homeostasis (Yin et al., 2016). Defects in this process have been associated with a variety of diseases, including neurodegenerative and inflammatory diseases or cancer (Galluzzi et al., 2015). Regarding cancer, it has been shown that some tumors with activating mutations in the RAS pathway, have elevated levels of autophagy, suggesting that autophagy inhibition in this type of cancers (lung, pancreas, melanoma, or colorectal cancer), could sensitize cells to death (Kinsey et al., 2019; Lee et al., 2019; Zhao and Zheng, 2019). In addition to cancer-driving mutations, there is evidence that extracellular cancer-promoting stimuli could increase autophagy and promote tumorigenesis. Leptin is a hormone-adipokine, synthesized and secreted mainly by adipose tissue. Its main function is to communicate the body’s energy status to the hypothalamus (Ahima and Osei, 2004). High levels of circulating leptin in obese women have been linked to the increased risk and poor prognosis of breast cancer (Hu et al., 2002; Garofalo et al., 2006; Wu et al., 2009). Leptin is considered as a pro-tumorigenic protein known to activate PI3K, JAK/STAT3, and MAPK signaling pathways (Hu et al., 2002; Cirillo et al., 2008). Furthermore, leptin can regulate processes such as inflammation, metabolism, and autophagy (Garcia-Robles et al., 2013; Delort et al., 2015; Park, 2018). Recent evidence has shown that leptin induces autophagy in breast (Nepal et al., 2015), colorectal (Malik et al., 2011), and osteosarcoma (Malik et al., 2011) cell lines, suggesting that autophagy has an important role in the pro-tumorigenic effects induced by leptin. It has been shown that triple negative breast cancer cells are more dependent on autophagy for survival than ER+ breast cancer cells, even under nutrient rich conditions (Maycotte et al., 2014). However, in the context of obesity where elevated levels of leptin are found, there is little evidence on the role of autophagy in breast cancer progression. Autophagy could have a synergistic or antagonistic role in the tumor promoting effects of leptin in breast cancer, therefore we evaluated the role of autophagy in leptin-induced proliferation and migration in breast cancer cell lines.

We found that leptin increased autophagy in ER+ MCF7 cells, but not in triple negative MDA-MB-231 cells. Leptin-induced autophagy was important for leptin-induced survival, proliferation, migration and ERK phosphorylation in MCF7 cells. While in MDA-MB-231, although autophagy was not induced by leptin treatment, autophagy inhibition decreased leptin-induced cell migration and ERK phosphorylation, indicating that basal autophagy in these cells contributed to leptin-induced migration and ERK phosphorylation. Our data indicates that autophagy inhibition could represent an alternative to relieve key events in breast tumor progression associated with elevated levels of leptin in obese individuals.

## Materials and Methods

### Cell Culture

The MCF7 cell line was maintained in Eagle’s Minimum Essential Medium (Eagle’s MEM) (Sigma, M0268) and the MDA-MB-231 cell line in Dulbecco’s Modified Eagle’s Medium/Nutrient blend F-12 Ham (DMEM/F12) (Caisson, DFP18), both supplemented with 10% fetal bovine serum (Biowest, BIO-S1650), under 5% CO_2_ atmosphere at 37°C.

### Leptin and Chloroquine Stimulation

The cells were synchronized by serum deprivation for 24 h. Then, MCF7 cells were treated with 400 ng/mL and MDA-MB-231 cells with 50 ng/mL of human recombinant leptin (Sigma, L4146) or vehicle (Control; Bovine serum albumin, 0.01%) for 24 h. For autophagic flux assays, 20 μM chloroquine, CQ (Sigma, C6628) was used for the last 2 h of treatment to inhibit autophagosomal degradation. For proliferation, migration and apoptosis assays, CQ was used at same concentration for 48 h.

### Protein Extraction

Cells were washed twice with PBS and subsequently lysed using RIPA buffer with protease inhibitors (Complete protease inhibitor cocktail, Sigma, 11697498001) and phosphatase inhibitors (PhosSTOP, Sigma, 4906845001). Cells were recovered by scraping and centrifuged at 12,000 rpm for 10 min at 4°C. The supernatant was recovered, and total proteins were quantified by Bradford’s method.

### Western Blotting

Protein lysates (30 μg) were separated in 12% SDS-polyacrylamide gels and transferred to PVDF membranes. The membranes were blocked with 5% skim milk in TBS-Tween buffer for 1 h and incubated with primary anti-LC3B (1:2000 Novus, NB100-2220), anti-SQSTM1/p62 (1:1000 Cell signaling, 88588), and anti-β-actin (1: 5000 Sigma-Aldrich, A5441) antibodies at 4°C overnight. Subsequently, three washes were performed with 0.05% TBS-Tween for 10 min and incubated in secondary anti-Mouse IgG antibody (1:20,000 Sigma, A2304) or anti-Rabbit IgG (1:20,000 Sigma, A0545) for 1 h at room temperature. Finally, immunodetection was performed with HRP-Western chemiluminescent Immobilon Substrate (Millipore, WBKLS0500) on C-DiGit transfer scanner (LI-COR). Relative intensity of bands was obtained by densitometric analysis of four independent experiments using Image J software.

### EGFP-LC3 Plasmid Transfection and Fluorescence Microscopy

150,000 cells were seeded in a 12 well-plate. Once attached, transfection of EGFP-LC3 plasmid (Addgene, 11546) was performed using Lipofectamine 3000 (Invitrogen, L3000015) according to the manufacturer’s instructions. 1.4 μg/μL of plasmid DNA were used and incubated overnight. Subsequently, cells were trypsinized and plated on coverslips. After 24 h of treatment, cells were fixed with 4% formaldehyde for 15 min and stained with Hoechst (10 μg/mL) for 5 min. Coverslips were mounted on slides using 50% glycerol in PBS as mounting media. Cytoplasmic EGFP-LC3 positive puncta indicated the formation of autophagosomes. Fluorescent images were obtained with an Axio Imager Zeiss microscope with a 63x objective. 30 cells were counted per experiment and graphs show the data from three independent experiments.

### Proliferation, Migration, Apoptosis and Cell Death Assays

To measure proliferation, 3,000 MCF7 and 2,500 MDA-MB-231 cells were plated in 96 multi-well plates. Proliferation was assessed in an IncuCyte ZOOM instrument as percent cellular confluence for 48 h. Migration was assessed by wound healing assay. 45,000 MCF7 or 30,000 MDA-MB-231 cells per well were plated in 96 well plates. 24 h after plating, the wound was made using an Essen WoundMaker. Cells were washed twice with PBS to remove unattached cells, cells were treated and observed during 48 h in an IncuCyte ZOOM instrument with a 4X objective or in an EVOS M7000 Thermo microscope. Wound healing percentage was calculated in the IncuCyte ZOOM analysis software or using ImageJ. Caspase 3/7 activity was evaluated using IncuCyte^®^ Caspase-3/7 Green Apoptosis Assay Reagent (Sartorius, 4440), a substrate with a caspase-3/7 recognition motif (DEVD). 3,000 MCF7 and 2,500 MDA-MB-231 cells were plated in 96-well plates, after 24 h, they were treated and IncuCyte^®^ Caspase-3/7 Green Apoptosis Assay Reagent (Sartorius 4440) was added to medium according to the manufacturer’s instructions. Caspase 3/7 activity was monitored in real time for 48 h. Propidium iodide (PI) staining was performed after 48 h of treatment. Cells were incubated with 1 μM PI for 10 min. and red fluorescence was evaluated in an IncuCyte ZOOM instrument. Caspase 3/7 activity or cell death are represented as percent confluency of green/red fluorescence normalized to total percent confluency as calculated by the IncuCyte ZOOM analysis software.

### Genetic Inhibition of Autophagy

shRNA pLKO.1 vectors were used for lentiviral transduction. Lentiviruses were prepared according to protocols published at the RNAi Consortium^1^. Briefly, Lenti-X 293T cells (Clontech) were transfected using TransIT-LT1 (Mírus MIR2300) transfection reagent with each pLKO.1 vector (TRCN000007857 or pLKO.1-SHC002), pMD2.g, pRRE, and pRSV packaging plasmids. Viruses were collected at 24 and 48 h, pooled and aliquots were frozen at −80°C. MCF7 or MDA-MB-231 cells were treated with polybrene (Sigma, 8 μg/mL) for 1 h before virus transduction. Cells were selected with puromycin (0.5 μg/mL) for 3 days, recovered and used for the indicated experiments.

### Statistical Analysis

Experimental data is shown as mean ± standard error mean (SEM). Statistical analysis was performed using ANOVA and Tukey test. *P* values less than 0.05 were considered statistically significant.

## Results

### Leptin Induces Autophagy in MCF7 but Not in MDA-MB-231 Cells

**FIGURE 1:**
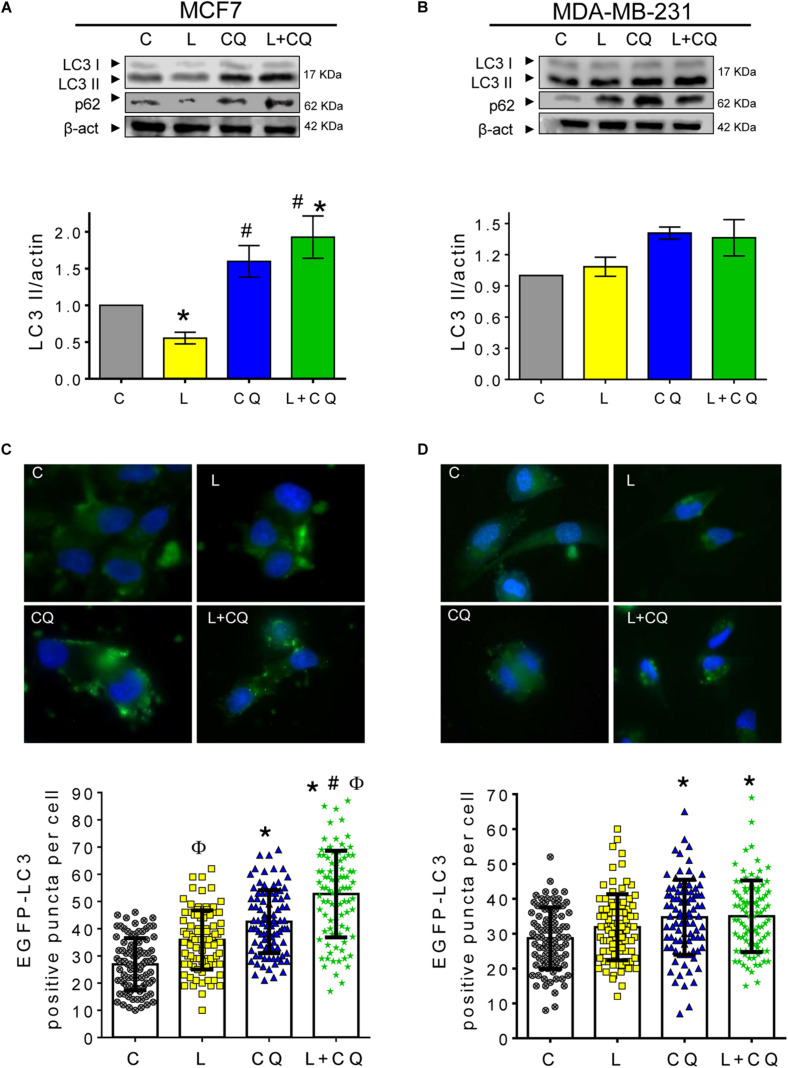
Leptin differentially induced autophagy in breast cancer cells. **(A,B)** Breast cancer cell lines were treated with leptin for 24 h ± CQ for the last 2 h and changes in autophagy markers LC3II and p62 were assessed by western blot or EGFP-LC3 positive puncta per cell were evaluated for autophagosome formation. Increased autophagic flux induced by leptin was observed in MCF7 cells by western blot **(A)** and EGFP-LC3 puncta **(C)** but not in MDA-MB-231 **(B,D)**. C, control treated with vehicle; L, leptin; CQ, chloroquine. Graphs show mean ± SEM; *n* = 4; *p* < 0.05. *vs. C; # vs. L; ϕ vs. CQ.

To evaluate the effect of leptin treatment on autophagy in MCF7 and MDA-MB-231 cells, the cells were treated with leptin or vehicle for 24 h and autophagic flux was determined by measuring the levels of LC3II and p62 proteins. During autophagy, LC3 is cleaved by ATG4 and conjugated to phosphatidylethanolamine forming LC3II. LC3II then integrates into the autophagosome double membrane and is used as a marker of autophagosome formation. LC3II abundance is related to the number of autophagosomes present in the cell (Klionsky et al., 2016), and it may be determined by western blot as LC3II migrates faster than LC3I in an SDS-PAGE. LC3II gets degraded upon autophagosome – lysosome fusion. Thus, the lysosomal inhibitor chloroquine (CQ) is used for autophagic flux determination since it prevents the binding of autophagosomes to the lysosome, inducing LC3II accumulation. Increased LC3II accumulation with CQ treatment along with a stimulus, when compared to LC3II levels in the stimuli by itself represents autophagic flux induction (Mizushima and Yoshimori, 2007). As shown in Figure 1A, in MCF7 cells leptin treatment results in a decrease in LC3II levels when compared to control cells (C). As expected, CQ treatment induced an increase in LC3II levels, and when the cells were treated with leptin + CQ, LC3II levels were further increased, indicating that leptin induces autophagic flux. In MDA-MB-231 cells, no differences in LC3II levels were observed in cells treated with leptin, CQ or leptin + CQ (Figure 1B). p62/SQSTM1 is an adaptor protein that binds LC3II and ubiquitinated proteins and organelles, targeting them to the autophagosome for degradation. As expected, CQ treatment induced p62 accumulation in both cell lines, indicating that CQ is effectively blocking autophagosome degradation. Leptin treatment decreased p62 levels in MCF7 cells when compared to vehicle treated cells, indicating autophagosomal degradation and autophagic flux induction. On the other hand, p62 levels increased after leptin treatment in MDA-MB-231 cells, and no further increase in p62 levels was observed when cells were treated with leptin + CQ (Figure 1B), suggesting that leptin might trigger a blocking effect on the degradation of autophagosomes. To further test the effects of leptin on autophagy, cells were transfected with an EGFP-LC3 expression construct to visualize autophagosomes by fluorescence microscopy. A change from a cytoplasmic diffuse pattern to a punctate staining of EGFP-LC3 indicates autophagosome formation. As expected, in MCF7 cells leptin significantly increased the number of autophagosomes compared to the control cells (L vs. C), and this was further increased in the in presence of CQ (Figure 1C; L + CQ vs. CQ). In contrast, in MDA-MB-231 cells, there was no significant increase in EGFP-LC3 positive puncta in leptin-treated cells compared to control cells (Figure 1D). These results indicate that leptin has a differential effect on autophagy induction in breast cancer cell lines. In MCF7 cells leptin induces autophagy, while in MDA-MB-231 cells leptin did not induce autophagy and might even have a blocking effect on basal autophagy levels as shown by increased p62 levels after leptin treatment.

### Autophagy Contributes to Leptin-Induced Proliferation in MCF7 but Not in MDA-MB-231 Cells

**FIGURE 2:**
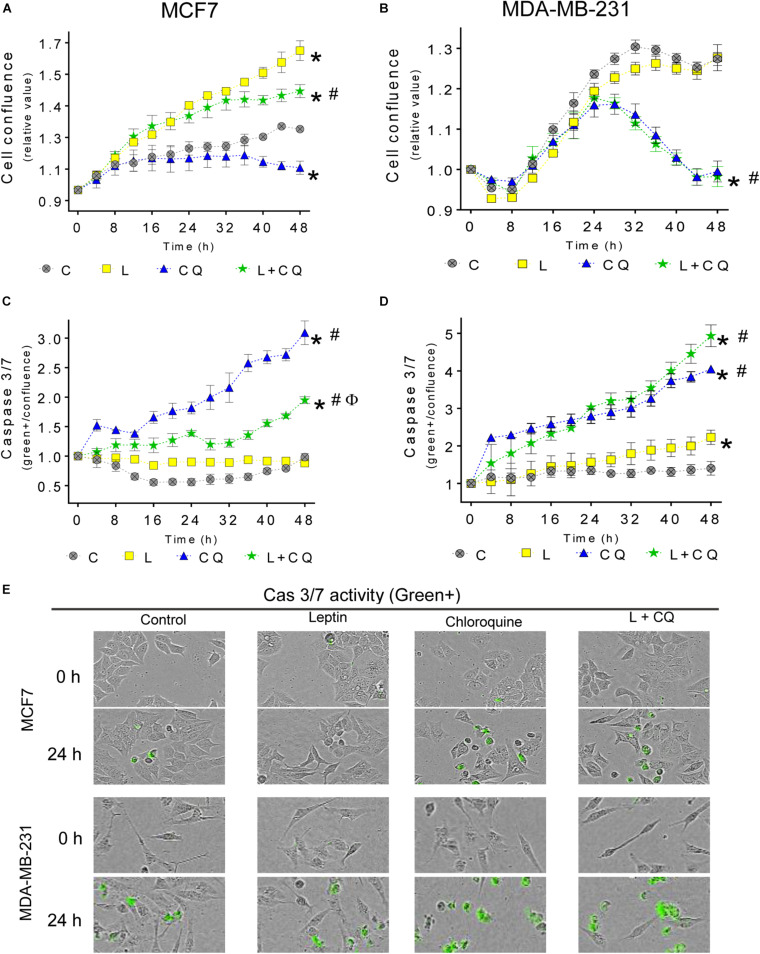
Autophagy contributes to leptin-induced proliferation in MCF7 but not in MDA-MB-231 cells. Cells were treated with leptin and/or CQ for 48 h. Leptin increased cell proliferation in MCF7 cells which was reduced by CQ treatment **(A)**. Apoptosis was increased by CQ in MCF7 cells and this was partly prevented by leptin treatment **(C)**. In MDA-MB-231 cells, leptin did not affect proliferation **(B)** and CQ treatment reduced proliferation **(B)** and increased apoptosis **(D)** independent of leptin treatment **(E)**. Representative images of caspase activity monitoring by green florescence with a caspase 3/7 substrate. Mean ± SEM; *n* = 5; *p* < 0.05. *vs. C; # vs. L; ϕ vs. CQ.

Next, we evaluated whether autophagy influences the proliferative and anti-apoptotic effects attributed to leptin. As shown in Figure 2, leptin induces proliferation in MCF7 cells, but not in MDA-MD-231 cells. CQ treatment alone, reduced the proliferation in both cell lines compared to control cells (CQ vs. C). In MCF7 cells, leptin increased proliferation even in the presence of CQ (Figure 2A). On the other hand, CQ treatment induced apoptosis in both cell lines (Figures 2C–E). Interestingly, in MCF7 cells, there was a significant decrease in apoptosis when the cells were treated with leptin + CQ compared with CQ treated cells, suggesting that leptin may protect MCF7 cells from apoptosis induced by autophagy inhibition. In contrast, in MDA-MB-231 cells, leptin had no effect on cellular proliferation (Figure 2B) but increased apoptosis, at least after 48 h (Figures 2D,E). When leptin was used in combination with CQ, proliferation and apoptosis levels were similar to CQ-treated cells (L + CQ vs. CQ), indicating that these effects were due to CQ and there was no significant effect induced by leptin treatment. These results suggest that MDA-MB-231 cells are more sensitive to CQ treatment and leptin does not influence this state as it does in MCF7 cells, in which leptin induces cellular proliferation and decreases CQ-induced apoptosis. Similar effects were observed in the MDA-MB-231 cell line when measuring total cell death with propidium iodide (PI) staining (Supplementary Figure 1).

### Autophagy Participates in Leptin-Induced Morphological Changes in Breast Cancer Cells

**FIGURE 3:**
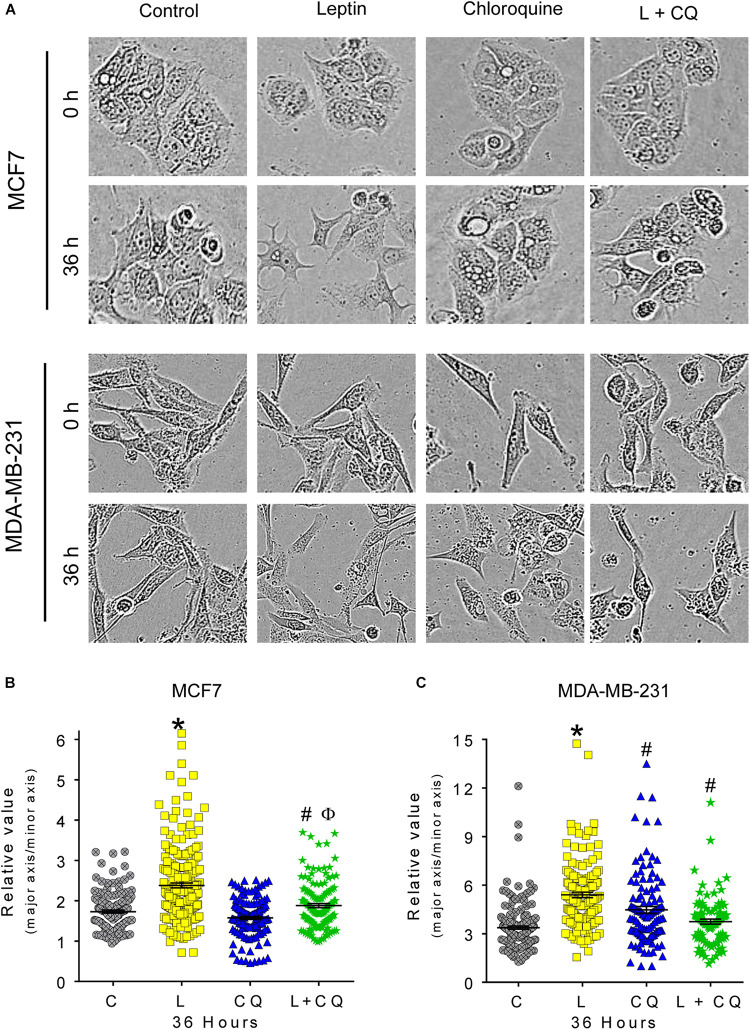
Autophagy participates in leptin-induced ETM-related changes in morphology of MCF7 and MDA-MB-231 cells. **(A)** Representative images of morphological changes in breast cancer cells. **(B,C)** Leptin favors mesenchymal spindle-like morphology in both cell lines. Autophagy inhibition reduced leptin-induced changes in morphology in both cell types. Mean ± SEM; *n* = 3; *p* < 0.05. *vs. C; # vs. L; ϕ vs. CQ.

Leptin is known to induce morphological changes associated to epithelial-mesenchymal transition (EMT). To evaluate these morphological changes, we performed a quantitative analysis considering two cell dimensions: major axis and minor axis. A ratio of 1 would represent a round shape; 1–2 epithelial morphology and greater than 2 spindle-like mesenchymal morphology (Supplementary Figure 2A). Cells with spindle-like morphology were evident after 24 h of leptin treatment (Supplementary Figure 2B), but changes in cell morphology were statistically significant at 36 h of treatment in both cell lines (Figure 3). Importantly, in the presence of CQ, leptin-induced spindle-like morphology was not observed in any of the cell lines (Figures 3B,C), indicating that autophagy contributes to the mesenchymal-like morphology promoted by leptin in breast cancer cells.

### Autophagy Contributes to Leptin-Induced Migration in Breast Cancer Cells

**FIGURE 4:**
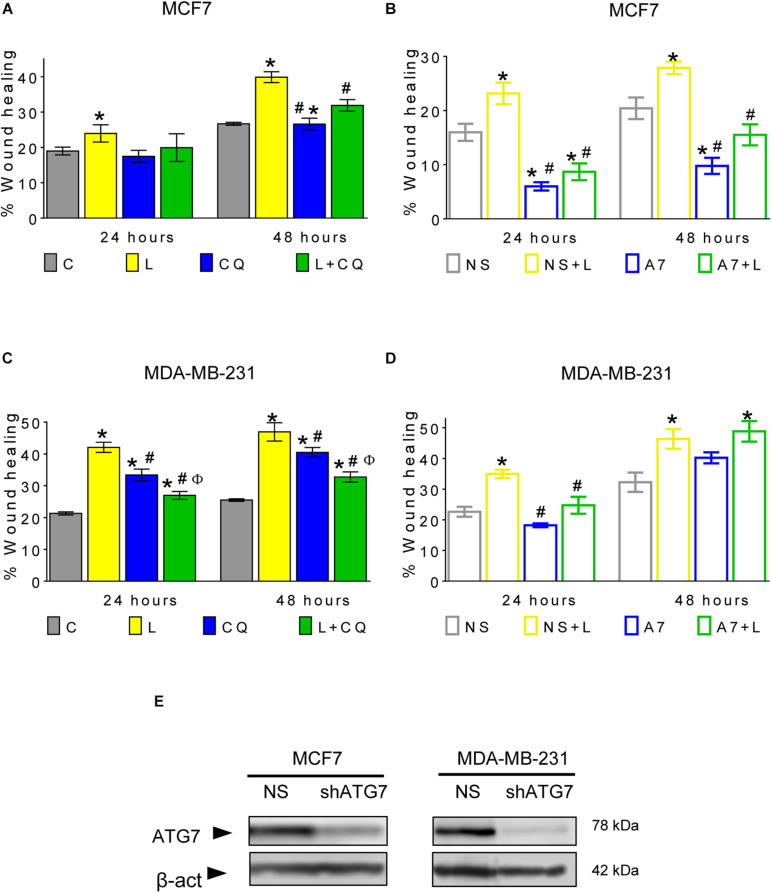
Autophagy contributes to leptin-induced migration in breast cancer cells. **(A–D)** Leptin increased cell migration and autophagy inhibition with CQ reduced leptin-induced migration **(A,C)**, in MCF7 at 48 h **(A)** and MDA-MB-231 cells at 24 and 48 h **(C)**. In both non-silencing cell lines, leptin increased the wound healing **(B,D)**. In MCF7 cells, genetic inhibition of autophagy with an ATG7 shRNA (A7) reduced leptin-induced migration in comparison with non-silencing cells (NS) at 48 h **(B)** while in MDA-MB-231 cells, leptin-induced migration was reduced at 24 h **(D)**. ATG7 shRNA similarly decreased the levels of ATG7 in both cell lines **(E)**. Mean ± SEM; wound healing with CQ *n* = 5; *p* < 0.05. *vs. C; # vs. L; ϕ vs. CQ. Wound healing with NS and shATG7 *n* = 4 for MCF7 cells; *n* = 3 for MDA-MB-231. *p* < 0.05. *vs. NS; # vs. NS + L.

A mesenchymal morphology is associated with increased migration capacities. Thus, we evaluated the effect of autophagy inhibition on leptin-induced cellular migration. As shown in Figure 4 and Supplementary Figure 2, leptin increased migration in a scratch-wound assay in both cell lines, and pharmacological inhibition of autophagy with CQ decreased migration at 24 and 48 h in MDA-MB-231 (Figure 4C) and at 48 h in MCF7 cells (Figure 4B), suggesting that autophagy plays an important role in leptin-induced migration in breast cancer cells. Importantly, in MDA-MB-231 cells, CQ treatment alone increased cell migration with respect to control (CQ vs. C; Figure 4C), indicating that the modulation of autophagy in MDA-MB-231 cells may have different effects on cell migration in the presence or absence of leptin.

To ensure that the effect on cell migration was due to the inhibition of autophagy and not to an autophagy-independent effect of CQ, cell migration was evaluated in cells with knockdown of the autophagy-related protein ATG7 (Figures 4B,D,E and Supplementary Figures 3, 5). In both cell lines expressing a non-silencing shRNA (NS), leptin increased wound healing. However, when ATG7 was knocked down (A7), leptin-induced migration was decreased in MCF7 cells at 24 and 48 h (Figure 4B and Supplementary Figure 3), while in MDA-MB-231 cells, migration was decreased only at 24 h (Figure 4D and Supplementary Figure 3). Apparently, MDA-MB-231 cells recover their leptin-induced migration ability after 48 h of ATG7 knockdown (Figure 4D), indicating that long term genetic autophagy inhibition does not limit the effect of leptin on cell migration in this cell line. Our data indicates that CQ decreased leptin-induced migration in both cell lines and this could also be observed by genetic modulation of autophagy at least at 24 h. Thus, autophagy is important for leptin-induced migration in both cell models (Figures 4A–D).

Additionally, we evaluated if the effect of autophagy on leptin-induced migration induced changes on EMT markers in breast cancer cells. However, no changes were found in the protein levels of E-cadherin, N-cadherin or vimentin (Supplementary Figure 4), suggesting that autophagy does not directly participate on the regulation of the protein levels of these adhesion proteins when cells are exposed to leptin.

### Autophagy Inhibition Decreases Leptin-Induced ERK Activation

**FIGURE 5:**
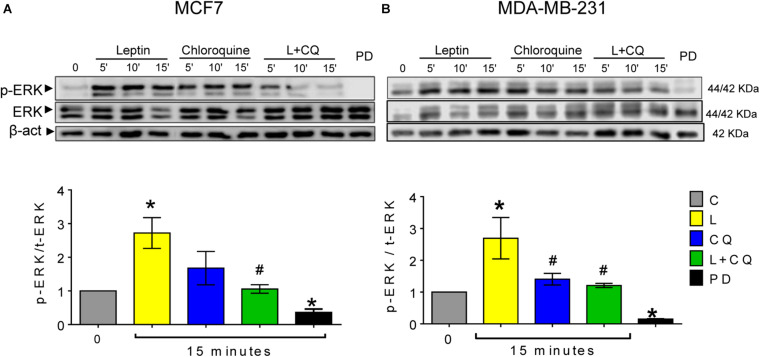
Autophagy inhibition decreased leptin-induced ERK activation in breast cancer cells. Leptin (L) induced ERK phosphorylation **(A,B)** in both cell lines. Autophagy inhibition with chloroquine (CQ) decreased leptin-induced ERK phosphorylation at 15 min. MEK inhibitor PD0325901 25 nM was used as a positive control. Mean ± SEM; *n* = 4; *p* < 0.05. *vs. C; # vs. L.

One of the pathways activated by leptin which is closely associated with cell migration is the MAPK pathway (Yin et al., 2004). We evaluated whether changes observed in cell migration were associated with the phosphorylation of ERK. As show in Figure 5, ERK phosphorylation was significantly increased in both cell lines after 15 min of leptin treatment. Autophagy inhibition with CQ decreased leptin-induced activation of ERK in both cell lines (Figure 5A), indicating an important role for autophagy in leptin-induced ERK phosphorylation, particularly for MCF7 cells where autophagy inhibition also decreased leptin-induced proliferation and migration.

## Discussion

Obesity has been associated with a higher risk and worse prognosis in different types of cancers, including breast cancer. In obese individuals, adipose tissue acts as a secretory organ of bioactive molecules, such as pro-inflammatory cytokines and adipokines, which are known to promote and support breast carcinogenesis (Dubois et al., 2014). Leptin and its OBRb receptor have been found to be overexpressed in breast tumors (Garofalo et al., 2006) and high serum or intratumoral levels of leptin have been associated with a poor prognosis in breast cancer (Wu et al., 2009), indicating an important role for this adipokine in breast tumor progression. Leptin activates signaling pathways such as PI3K, JAK/STAT3, and MAPK which play key roles in cellular proliferation (Yin et al., 2004; Dubois et al., 2014), migration (Ghasemi et al., 2019), invasion (Olea-Flores et al., 2018), and angiogenesis (Modzelewska et al., 2019). Leptin has also been proposed to regulate autophagy (Park, 2018), a process closely related to tumor progression. In breast cancer cells, leptin has been shown to induce the expression of autophagy-related proteins ATG5 and BECLIN-1 through p53/FoxO3A (Nepal et al., 2015) and/or the estrogen receptor/AMPK/FoxO3A axis (Raut et al., 2017), and to regulate cellular proliferation. Thus, since leptin has been shown to affect autophagy, modulation of autophagy should affect the pro-tumoral effects of leptin. In this work, we show that leptin has a different effect on autophagy in breast cancer cell lines with different phenotypes. In MCF7 (ER+) cells, leptin induced autophagy, while in MDA-MB-231 cells (triple negative) autophagy was not induced by leptin treatment.

Signaling from RAS/MAPK, STAT3, and p53 pathways participate in the regulation of autophagy (Nepal et al., 2015). PI3K/AKT negatively regulates autophagy in response to grown factor signaling (Wang et al., 2019) by phosphorylating mTOR, which in turn phosphorylates and inactivates the ULK1 kinase, responsible for the initiation of autophagy (Xu et al., 2020). The RAS/RAF/MEK/ERK and STAT3 pathways are also known to be intimately related to autophagy, although their relationship is not so straightforward. Cancer cells with activating mutations in RAS have high levels of autophagy, necessary for metabolic maintenance and oncogenic transformation (Guo et al., 2011; Lock et al., 2011) but inhibition of MEK has also been shown to activate the AMPK/ULK1 signaling axis and induce autophagy in RAS mutated cancers (Kinsey et al., 2019). Autophagy has also been related to the regulation of the JAK/STAT3 pathway and vice versa. Autophagy was necessary for STAT3 signaling in triple negative breast cancer cells (Maycotte et al., 2014) and in starved cancer cells (Yoon et al., 2010). Also, STAT3 has been shown to inhibit autophagy, since STAT3 inhibitors can be potent inducers of autophagy in cancer cells (Shen et al., 2012). Therefore, the signaling pathways activated by leptin could have a direct influence on autophagy and the activation of autophagy could also possibly act as a feedback mechanism for the maintenance of cancer-related leptin signaling. Our data suggests that leptin has different effects on the modulation of autophagy depending on the cellular context and mutational background. In this regard, MDA-MB-231 cells are known to have activating mutations in the RAS pathway (KRAS, BRAF), high levels of activated STAT3 (Maycotte et al., 2014), and missense mutations in p53 (Harvard, 2020). Thus, we suggest that in MDA-MB-231 cells, leptin did not induce autophagy due to its mutational background and the high constitutive levels of activation of MAPK and STAT3 signaling pathways (Marotta et al., 2011; Maycotte et al., 2014). On the other hand, MCF7 cells do not present activating mutations in genes related to RAS or STAT3 signaling pathways and express wild type p53. In these cells, autophagy was induced by leptin treatment, suggesting a possible role for leptin-induced activation of RAS, STAT3, or p53 signaling pathway in the induction of autophagy. Although differential effects could also be due to different levels of the leptin receptor, it has previously been reported that both cell lines used in this investigation have similar levels of OBRb (Yom et al., 2013) and similar concentrations of leptin used have been shown to have effects on migration and metalloprotease secretion in the same breast cancer cell lines (Juarez-Cruz et al., 2019). Importantly, although the cell lines used in this study are extensively used as *in vitro* breast cancer models, our data does not exclude the possibility that the effects observed are particular to these cell lines and might not be a general mechanism occurring in other cell lines from the corresponding breast cancer subtype. However, our data shows that leptin can have differential effects on the induction of autophagy in different breast cancer cells and we suggest that different effects might be due to the cancer cell mutational background.

In cancer, autophagy is recognized as an event that allows cancer cell survival and adaptation to stress conditions occurring within the tumor microenvironment and during metastasis (White et al., 2015). After the identification of tumors that depend on autophagy for survival (Levy et al., 2017; Kinsey et al., 2019), the inhibition of autophagy has been proposed as a therapeutic strategy in diverse cancer types by using CQ or its analog hydroxychloroquine (HCQ) to block autophagy in combination with cancer therapy (Levy et al., 2017). In this work, we found that the inhibition of autophagy decreased leptin-induced proliferation, migration, ERK phosphorylation and morphological changes in MCF7 cells; while in MDA-MB-231 cells, inhibition of autophagy decreased leptin-induced mesenchymal-related morphological changes, migration and ERK phosphorylation. In MCF7 cells, it has previously been shown that leptin induced cell proliferation (Yin et al., 2004), prevented apoptosis (Perera et al., 2008), induced mesenchymal-like morphological changes (Mishra et al., 2017), and induced cellular migration (Yuan et al., 2014; Juarez-Cruz et al., 2019). We confirmed these findings and demonstrated that leptin-induced autophagy is involved in the cancer-promoting features induced by leptin (Figures 2–4). In MDA-MB-231 triple negative breast cancer cells, which are known to have a high invasive capacity, leptin did not induce autophagy (Figure 1) neither induced changes in proliferation or apoptosis (Figure 2), but increased cell migration, in agreement with previous reports (Dubois et al., 2014; Weichhaus et al., 2014; Juarez-Cruz et al., 2019). In these cells, pharmacological inhibition of autophagy decreased proliferation independent of leptin treatment and increased cell death (Figure 2 and Supplementary Figure 1), indicating that basal levels of autophagy have an important role for the maintenance of cell survival and proliferation in this cell line. In agreement with this observation, triple negative breast cancer cells have been shown to be dependent on autophagy for survival, that is, autophagy inhibition induces cell death (Maycotte et al., 2014). Importantly, combined L + CQ treatment slightly increased apoptosis in the MDA-MB-231 cell line, probably reflecting an enhanced inhibition of autophagy since leptin blocked autophagy in this cell line (Figure 1). Importantly, leptin-induced migration was decreased after pharmacological or genetic inhibition of autophagy in the MDA-MB-231 cell line (Figure 4) suggesting that although leptin did not induce autophagy in these cells, basal levels of autophagy contribute to leptin-induced migration.

Leptin-induced mesenchymal morphology and cell migration were reduced by pharmacological or genetic inhibition of autophagy in both cell lines, suggesting that autophagy regulates signaling pathways involved in this phenotype. Although we did not find changes in EMT-related proteins (Supplementary Figure 4), spindle-like changes in cell morphology induced by leptin suggests that either leptin primes the cells to undergo EMT or that changes in EMT protein expression occur at longer exposure times. Nevertheless, these changes in morphology were related to increased cell motility in both cell lines. One of the most important pathways that regulates focal adhesion dynamics during cell motility is the MAPK pathway through ERK activation (Olea-Flores et al., 2018). Since ERK is known to be activated by leptin, we assessed changes in ERK phosphorylation after the inhibition of autophagy. As expected, leptin increased the phosphorylation of ERK in the MCF7 cell line and further increased ERK phosphorylation in the MDA-MB-231 cell line. Leptin-induced ERK phosphorylation was reduced by the inhibition of autophagy with CQ treatment (Figure 5). Interestingly CQ treatment alone increased ERK phosphorylation in both cell lines. We propose that the inhibition of autophagy can activate ERK signaling due to the accumulation of reactive oxygen species (ROS). We and others have shown that ROS accumulate after the inhibition of autophagy (Cotzomi-Ortega et al., 2020), mostly due to the accumulation of damaged mitochondria (Azad et al., 2009; Lee et al., 2016), and ROS have been closely related to the activation of MAPK signaling pathways (Son et al., 2011) but this remains to be tested in future studies. Importantly, combined L + CQ treatment decreased ERK phosphorylation, indicating an important role for autophagy in maintaining leptin-induced ERK activation. The role of autophagy on the activation of other signaling pathways activated by leptin remains to be evaluated. In this regard, autophagy has been related to ERK activation, since ATG proteins, LC3II, and p-ERK have been found to colocalize on the autophagosome membrane and it has been suggested that ATG proteins serve as scaffolds that coordinate the activation of the RAF/MEK/ERK signaling cascade on the autophagosome membrane (Martinez-Lopez et al., 2013). Additionally, it has also been proposed that autophagy can function as a regulator of focal adhesions. In this regard, autophagy has been shown to facilitate the cellular turnover of focal adhesion complexes that lead to cellular motility (Kenific et al., 2016a,b) and small GTPases that participate in focal adhesions such as RAC1, have been also been proposed to be substrates for autophagy (Carroll et al., 2013). Thus, autophagy could regulate leptin-induced cell migration directly, by modulating focal adhesion complex turnover, or indirectly, through ERK phosphorylation.

Thus, we demonstrate that leptin induced autophagy in the non-metastatic ER + MCF7 cell line, and this process contributes to leptin-induced proliferation, migration and ERK phosphorylation. In the highly metastatic triple negative MDA-MB-231 cells, leptin did not induce autophagy, but basal autophagy inhibition decreased proliferation independent of leptin treatment, increased apoptosis and decreased leptin-induced cellular migration and ERK phosphorylation. Despite having different effects on autophagy induction in both breast cancer cell lines, autophagy contributed to leptin-induced migration and ERK phosphorylation and this effect could be due to leptin-induced autophagy in MCF7 cells and to basal autophagic levels in the MDA-MB-231 cell line. Since autophagy inhibition has been considered as a potential cancer therapy in recent years, our data supports the potential use of autophagy inhibition in breast cancer, particularly for obese breast cancer patients with high levels of circulating leptin which are known to have an unfavorable prognosis with current therapeutic strategies.

## Data Availability Statement

The raw data supporting the conclusions of this article will be made available by the authors, without undue reservation.

## Author Contributions

AG-M, PM, and EC-S contributed to the conception and design of the study. AG-M, KS-A, JM-A, and AR-C performed the experiments. AG-M, PM, JR-L, NN-T, and EC-S contributed to data analysis and writing of the manuscript. All authors contributed to critical analysis and approval of the final version of the manuscript.

## Conflict of Interest

The authors declare that the research was conducted in the absence of any commercial or financial relationships that could be construed as a potential conflict of interest.

## Footnotes

1 http://www.broadinstitute.org/rnai/trc/lib
