# Supplementary material for: Autophagy Mediates Leptin-Induced Migration and ERK Activation in Breast Cancer Cells
Source: Front Cell Dev Biol. 2021 Mar 8;9:644851. doi: 10.3389/fcell.2021.644851 (PMC7982408; doi:10.3389/fcell.2021.644851)
Supplement: Supplementary file 1 [file Data_Sheet_1.PDF]

Supplementary Material

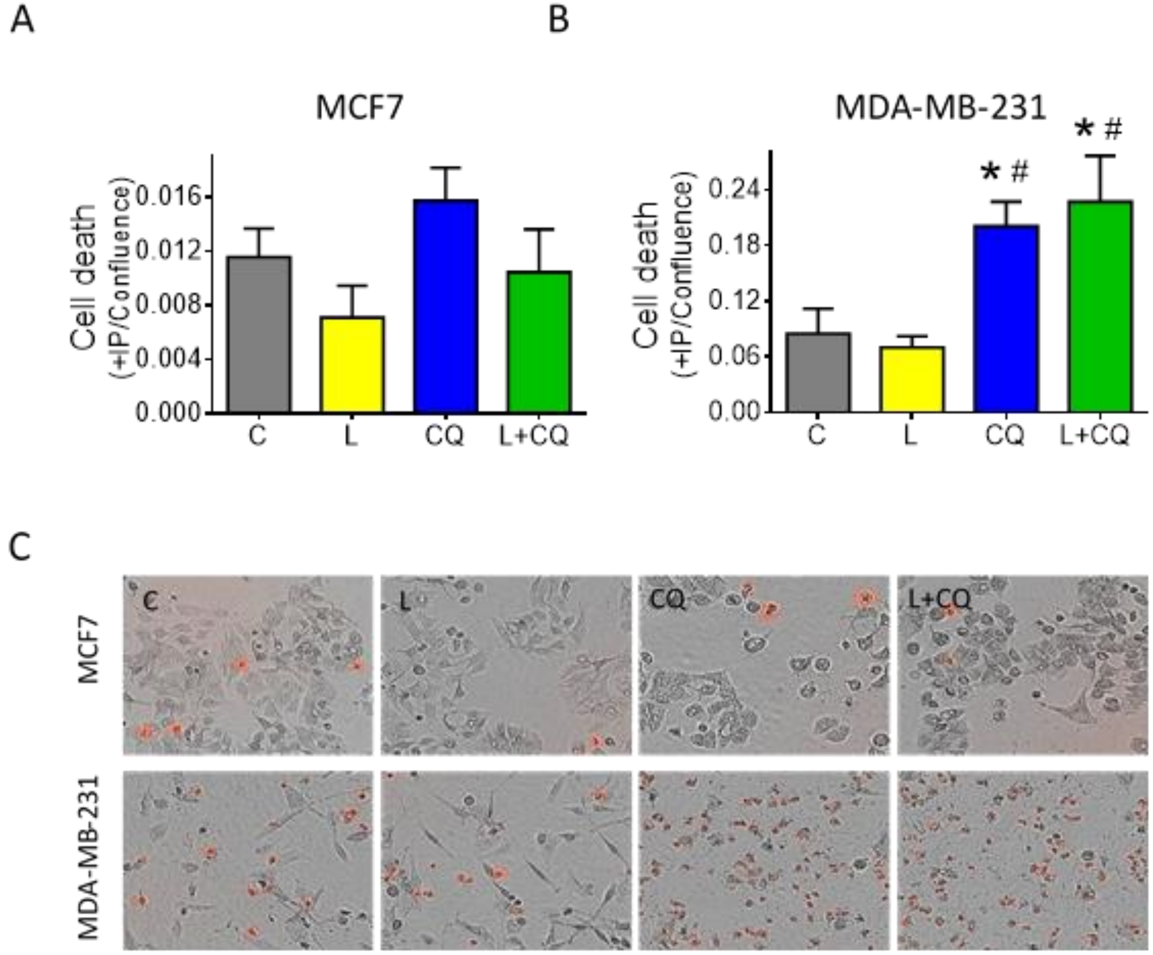

Figure S1. Effect of leptin and autophagy inhibition on breast cancer cell death. 48 h after treatment, cells were incubated with propidium iodide (PI) to evaluate cell death. (A) In MCF7 cells, the different treatments did not induce significant differences in PI staining. (B) In MDA-MB-231 cells, treatments containing CQ significantly increased PI staining, similar to the effect observed on cell proliferation. (C) Representative images of IP staining. 20X objective. Mean ± SEM; n=5; $p<0.05$. #vs L,*vs C.

A

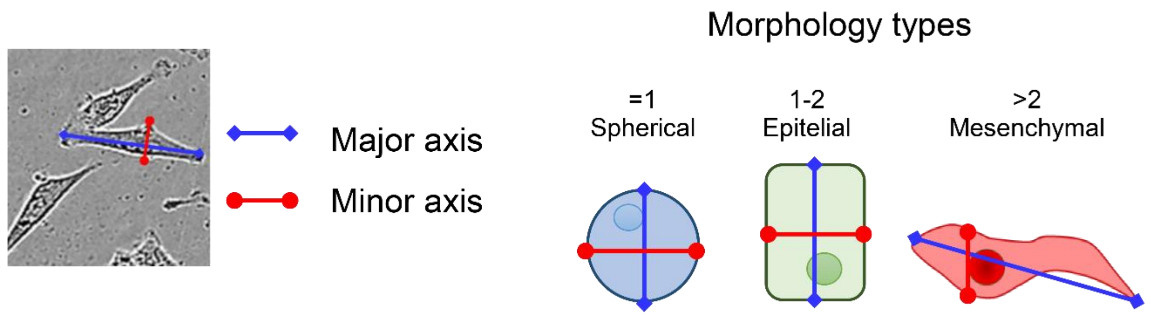

B

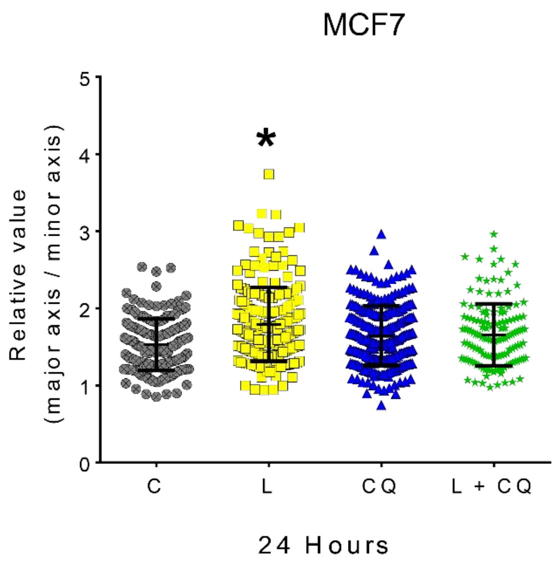

Figure S2. Effect of leptin and autophagy inhibition on MCF7 cell morphology. (A) Methodological strategy for the determination of cell morphology. The axis considered for the major axis/ minor axis relationship are shown and the ratio correspondence with the type of cell morphology. (B) At 24 hours of treatment with leptin or CQ, significant differences were observed only in cells treated with leptin (L) where cells acquire a mesenchymal-like morphology, these changes were prevented with chloroquine (L+CQ) treatment. Mean ± SEM; n=3; $p<0.05$. *vs C.

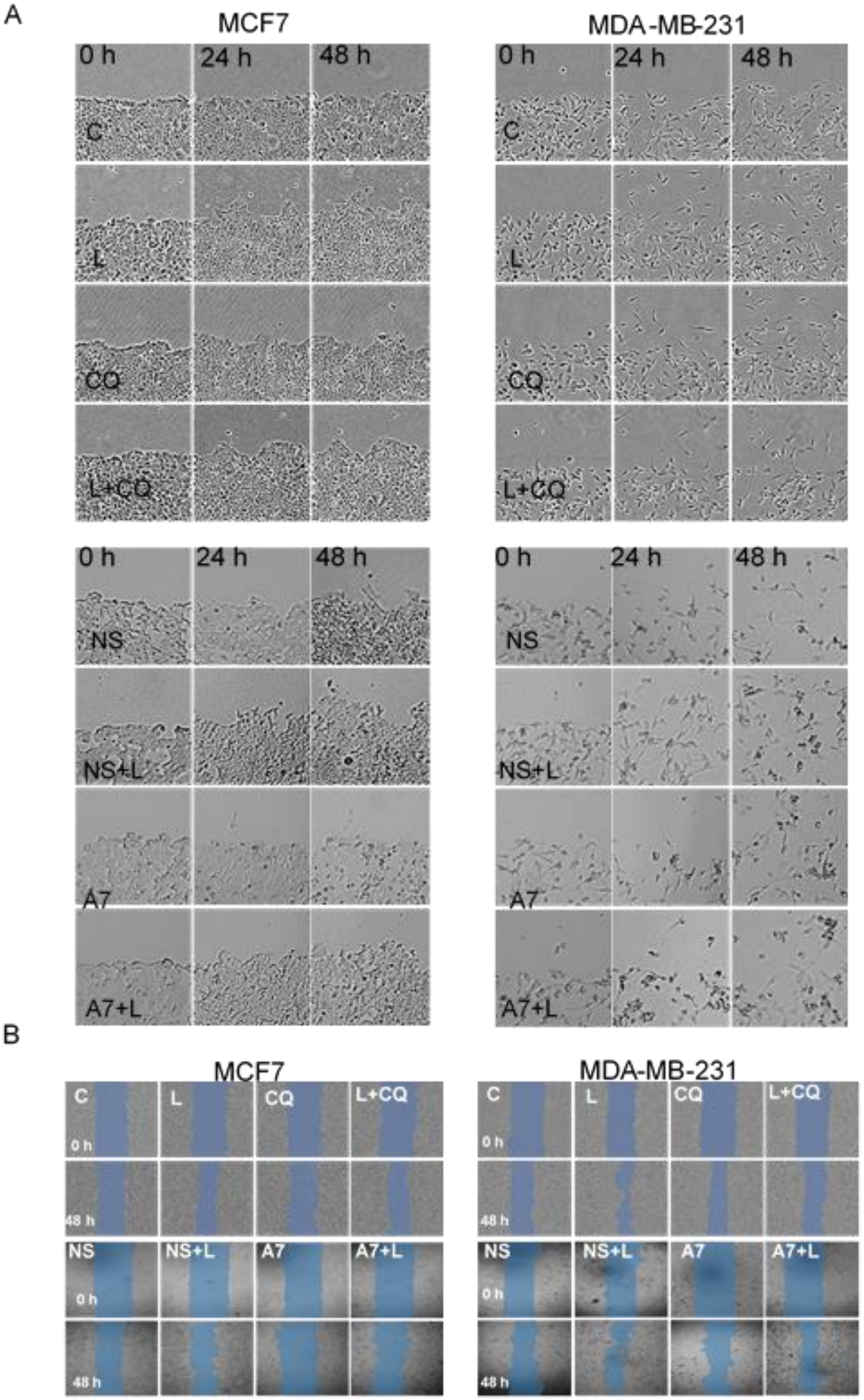

Figure S3. Representative images of wound healing assay on breast cancer cell lines. (A) Representative images of cell migration in front of the wound in each condition. (B) In both cells lines, leptin (L) increased wound healing which was prevented by autophagy inhibition with CQ. In same way, genetic inhibition of autophagy with an ATG7 shRNA (A7) reduced leptin-induced wound healing in comparison with non-silencing cells (NS).

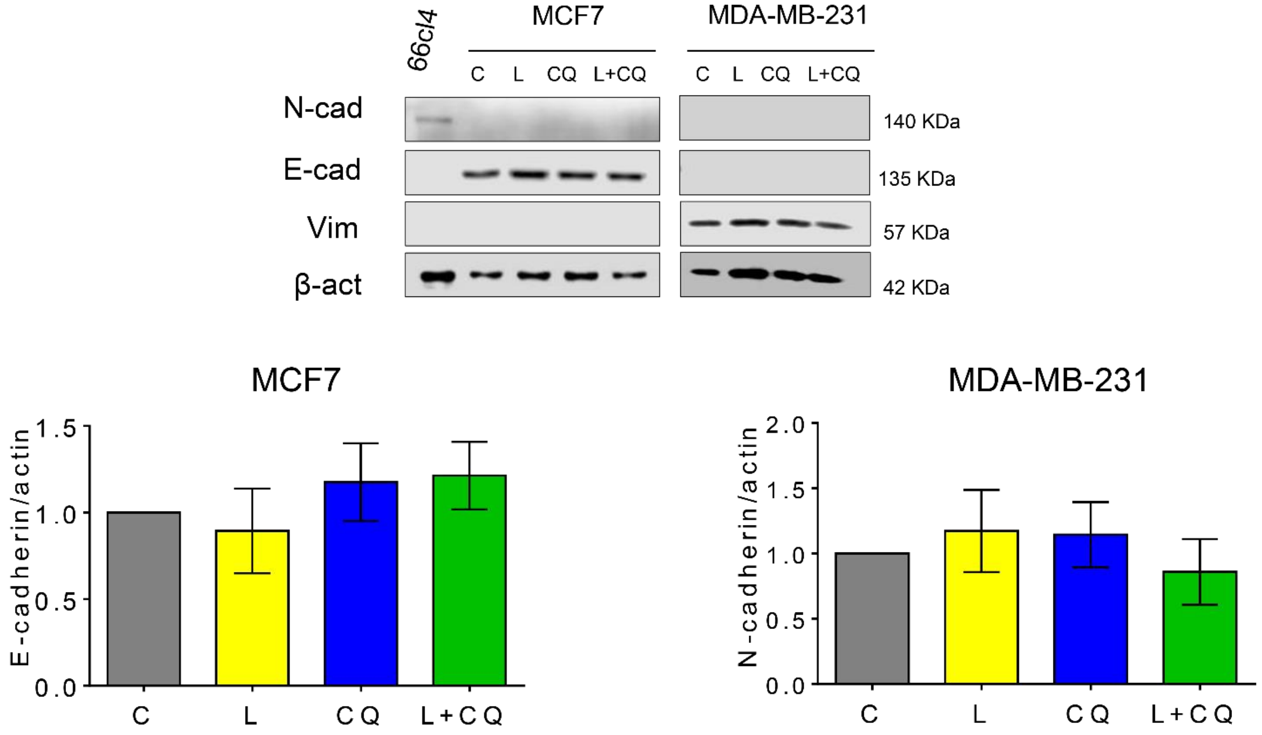

Figure S4. Effect of leptin (L) and autophagy inhibition with chloroquine (CQ) on EMT markers in breast cancer cells. No differences were observed in the presence of EMT markers. Mean ± SEM; n=4; $p<0.05$.

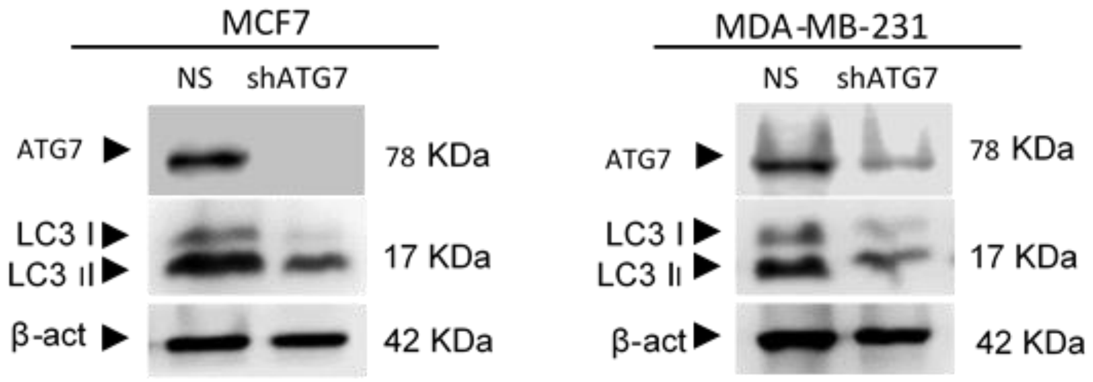

Figure S5. Validation of ATG7 silencing by Western blotting and its effect on LC3 processing in the breast cancer cell lines used in this study. ATG7 silencing by shRNAs decreased LC3 processing to LC3 II when compared with non-silencing cells (NS).
